# Supplementary material for: Temporal patterns of multiple long-term conditions in individuals with intellectual disability living in Wales: an unsupervised clustering approach to disease trajectories
Source: Front Digit Health. 2025 Mar 27;7:1528882. doi: 10.3389/fdgth.2025.1528882 (PMC11983499; doi:10.3389/fdgth.2025.1528882)
Supplement: Supplementary file 1 [file Datasheet1.pdf]

Supplementary Material

SUPPLEMENTARY TABLES

Table S1: Long-term conditions used in the study and their system categorisation.

Condition	Abbreviated Name	Included Conditions	Condition Category
Addisons Disease			Endocrine
Anaemia			Blood
Barretts Oesophagus			Digestive
Bronchiectasis			Respiratory
Cancer			Neoplasms
Cardiac Arrhythmias		Atrial Fibrillation, Atrial Flutter, Supraventricular Tachycardia, Heart Block	Circulatory
Cerebral Palsy			Nervous
Chronic Airway Diseases	Chr. Airway Diseases	Eosinophil Bronchitis, Chronic Obstructive Pulmonary Disease (COPD), Asthma	Respiratory
Chronic Arthritis	Chr. Arthritis	Osteoarthritis, Arthritis, Autoimmune Arthritis	Musculoskeletal
Chronic Constipation	Chr. Constipation		Digestive
Chronic Diarrhoea	Chr. Diarrhoea		Digestive
Chronic Kidney Disease	CKD		Genitourinary
Chronic Pain Conditions	Chr. Pain Conditions	Fibromyalgia, Pelvic Pain, Chronic Back Pain	Musculoskeletal
Chronic Pneumonia	Chr. Pneumonia		Respiratory
Cirrhosis			Digestive
Coronary Heart Disease	CHD		Circulatory
Dementia			Mental
Diabetes			Endocrine
Dysphagia			Digestive
Epilepsy			Nervous
Heart Failure			Circulatory
Hearing Loss			Ear
Hypertension			Circulatory
Inflammatory Bowel Disease	IBD		Digestive
Insomnia			Nervous
Interstitial Lung Disease		Interstitial Pulmonary Fibrosis	Respiratory

Continued on next page

Table S1 – *Continued from previous page*

Condition	Abbreviated Name	Included Conditions	Category
Mental Illness		Schizophrenia, Severe Depression	Mental
Menopausal And Perimenopausal			Genitourinary
Multiple Sclerosis	MS		Nervous
Neuropathic Pain			Nervous
Osteoporosis			Musculoskeletal
Parkinsons			Nervous
Peripheral Vascular Disease	PVD	Peripheral Arterial Disease	Circulatory
Polycystic Ovary Syndrome	PCO		Endocrine
Psoriasis			Skin
Reflux Disorders		Gastro-oesophageal reflux disease (GORD), Gastric and Peptic Ulcer Diseases, Helicobacter Pylori, Dyspepsia	Digestive
Stroke			Nervous
Thyroid Disorders		Hyperthyroidism, Hypoparathyroidism, All Hypo and Hyper, Hashimoto	Endocrine
Tourette			Mental
Visual Impairment			Eye

Table S2: Number of individuals with 1 to ≥ 6 conditions by sex.

Number of conditions	All Patients	Males	Females
1 condition	1838	1073	765
2 conditions	2104	1261	843
3 conditions	1955	1056	899
4 conditions	1784	936	848
5 conditions	1393	718	675
6+ conditions	3995	1786	2209

Table S3: Distribution of males and females across age, ethnicities, and deprivation quintiles groups.

**Category**	**Males**	**Females**
**Total**	6830	6239
**Age, years**		
<45	3010	2496
$\geq$ 45	3820	3743
**Ethnic group**		
Asian	104	75
Black	26	17
Mixed	9	6
Other	16	17
Unknown	1904	1734
White	4771	4390
**WIMD 2019 Quintile**		
1. Most deprived	1659	1536
2	1345	1237
3	1084	1003
4	1022	927
5. Least deprived	679	667

Table S4: Condition counts for all males and by age group.

Condition	All Males		Males < 45		Males ≥ 45	
	Count	%	Count	%	Count	%
Mental Illness	2239	32.8	1054	35.0	1185	31.0
Epilepsy	2120	31.0	1062	35.3	1058	27.7
Reflux Disorders	2027	29.7	894	29.7	1133	29.7
Hypertension	1602	23.5	467	15.5	1135	29.7
Chr. Airway Diseases	1597	23.4	690	22.9	907	23.7
Diabetes	1383	20.2	415	13.8	968	25.3
CKD	1342	19.6	313	10.4	1029	26.9
Chr. Arthritis	1264	18.5	331	11.0	933	24.4
Hearing Loss	1140	16.7	394	13.1	746	19.5
Insomnia	1078	15.8	520	17.3	558	14.6
Thyroid Disorders	961	14.1	366	12.2	595	15.6
Anaemia	953	14.0	235	7.8	718	18.8
Cardiac Arrhythmias	899	13.2	196	6.5	703	18.4
Dysphagia	833	12.2	249	8.3	584	15.3
IBD	778	11.4	315	10.5	463	12.1
CHD	671	9.8	88	2.9	583	15.3
Cancer	666	9.8	134	4.5	532	13.9
Dementia	607	8.9	43	1.4	564	14.8
Chr. Pain Conditions	598	8.8	280	9.3	318	8.3
Neuropathic Pain	584	8.6	237	7.9	347	9.1
Stroke	584	8.6	102	3.4	482	12.6
Cerebral Palsy	574	8.4	320	10.6	254	6.6
Heart Failure	554	8.1	83	2.8	471	12.3
Chr. Constipation	534	7.8	176	5.8	358	9.4
Chr. Diarrhoea	420	6.1	158	5.2	262	6.9
Visual Impairment	411	6.0	123	4.1	288	7.5
Osteoporosis	409	6.0	67	2.2	342	9.0
PVD	393	5.8	76	2.5	317	8.3
Psoriasis	350	5.1	164	5.4	186	4.9
Chr. Pneumonia	336	4.9	71	2.4	265	6.9
Parkinson's	131	1.9	15	0.5	116	3.0
Barrett's Oesophagus	108	1.6	36	1.2	72	1.9
Cirrhosis	86	1.3	33	1.1	53	1.4
Bronchiectasis	78	1.1	22	0.7	56	1.5
Interstitial Lung Disease	54	0.8	6	0.2	48	1.3
Tourette	30	0.4	17	0.6	13	0.3
Addison's Disease	17	0.2	9	0.3	8	0.2
MS	10	0.1	5	0.1	5	0.1

Table S5: Condition Counts for all females and by age group.

Condition	All Females		Females < 45		Females ≥ 45	
	Count	%	Count	%	Count	%
Mental Illness	2188	35.1	932	37.3	1256	33.6
Thyroid Disorders	1921	30.8	676	27.1	1245	33.3
Reflux Disorders	1876	30.1	772	30.9	1104	29.5
Epilepsy	1766	28.3	781	31.3	985	26.3
Chr. Airway Diseases	1698	27.2	714	28.6	984	26.3
Hypertension	1496	23.9	298	11.9	1194	31.9
Chr. Arthritis	1471	23.6	300	12.0	1171	31.3
CKD	1313	21.0	286	11.5	1027	27.4
Anaemia	1285	20.6	462	18.5	823	22.0
Diabetes	1217	19.5	342	13.7	875	23.4
Menopausal & Perimenopausal	1185	19.1	219	8.8	970	25.9
Hearing Loss	1060	17.0	367	14.7	693	18.5
Chr. Pain Conditions	970	15.5	468	18.8	502	13.4
Insomnia	933	15.0	410	16.4	523	14.0
Dysphagia	803	12.9	248	9.9	555	14.8
IBD	798	12.8	303	12.1	495	13.2
Cancer	786	12.5	148	5.8	634	16.9
Cardiac Arrhythmias	766	12.3	148	5.8	620	16.6
Neuropathic Pain	725	11.6	261	10.5	464	12.4
Dementia	719	11.5	47	1.9	672	18.0
Osteoporosis	694	11.1	117	4.7	577	15.4
Chr. Constipation	579	9.3	231	9.3	348	9.3
Stroke	566	9.1	68	2.7	498	13.3
Heart Failure	496	7.9	56	2.2	440	11.8
Cerebral Palsy	489	7.8	244	9.8	245	6.5
CHD	476	7.6	46	1.8	430	11.5
Chr. Diarrhoea	435	7.0	171	6.9	264	7.1
Visual Impairment	426	6.8	114	4.6	312	8.3
PVD	389	6.2	61	2.4	328	8.8
Psoriasis	375	6.0	157	6.3	218	5.8
Chr. Pneumonia	332	5.3	98	3.9	234	6.3
Parkinson's	136	2.2	17	0.7	119	3.2
Barrett's Oesophagus	110	1.8	27	1.1	83	2.2
Bronchiectasis	106	1.7	31	1.2	75	2.0
Cirrhosis	77	1.2	26	1.0	51	1.4
Interstitial Lung Disease	57	0.9	9	0.4	48	1.3
Tourette	16	0.3	10	0.4	6	0.2
Addison's Disease	14	0.2	5	0.2	9	0.2

Continued on next page

Table S5 continued

Condition	All Females		Females < 45		Females ≥ 45	
	Count	%	Count	%	Count	%
MS	12	0.2	6	0.2	6	0.2

Table S6: Comparison of Calinski-Harabasz scores for different numbers of clusters in spectral clustering

	Number of clusters								
	2	3	4	5	6	7	8	9	10
Males									
<45 years	13.7	9.6	6.1	6.2	4.8	4.4	3.2	3.8	3.3
≥ 45 years	82.1	103.8	66.2	62.2	46.1	45.8	42.9	41.9	38.2
Females									
<45 years	15.1	14.8	6.5	6.2	5.8	4.9	4.2	4.8	4.2
≥ 45 years	116.3	115.9	98.9	77.4	70.4	62.2	57.0	47.8	46.5

Table S7: Number of trajectories (N traj) considered in the clustering algorithm for a trajectory length of three, stratified by sex and age group. Only trajectories with a minimum of ten patients were included.

N traj	Males	Females
<45 years	37	88
≥45 years	229	439

Table S8: Condition percentages of the trajectories included in clusters for males, stratified by age groups. Each cluster presents the most frequent conditions including in the trajectories alongside the count of trajectories (N traj) and total patient numbers (N patients). Percentages (%) are calculated based on the total number of trajectories in each cluster. Clusters are presented in descending order of patient count within each age category.

N traj	N patients	Condition percentages and clusters
**a. Males $<$ 45 years**		
37	549	chr. pain conditions (37.8%), mental illness (37.8%), insomnia (37.8%), reflux disorders (35.1%), epilepsy (27.0%), chr. airway diseases (21.6%), chr. arthritis (21.6%), neuropathic pain (18.9%), cerebral palsy (13.5%), dysphagia (10.8%), hypertension (10.8%), diabetes (8.1%), CKD (5.4%), chr. constipation (5.4%), anaemia (5.4%)
**b. Males $\geq$ 45 years**		
112	2824	CHD (44.6%), CKD (35.7%), cardiac arrhythmias (34.8%), heart failure (32.1%), diabetes (24.1%), hypertension (24.1%), anaemia (21.4%), PVD (19.6%), chr. airway diseases (15.2%), chr. arthritis (13.4%), stroke (7.1%), reflux disorders (6.3%), chr. pneumonia (6.3%), Cancer (5.4%)
81	1557	reflux disorders (53.1%), chr. arthritis (50.6%), insomnia (29.6%), chr. airway diseases (25.9%), neuropathic pain (24.7%), chr. pain conditions (23.5%), CHD (17.3%), anaemia (16.0%), mental illness (12.3%), cardiac arrhythmias (9.9%), heart failure (9.9%), hearing loss (8.6%)
36	633	dysphagia (50.0%), chr. constipation (41.7%), chr. pneumonia (36.1%), epilepsy (27.8%), dementia (27.8%), mental illness (19.4%), reflux disorders (13.9%), CKD (13.9%), chr. diarrhoea (11.1%), anaemia (8.3%), chr. airway diseases (5.6%), cardiac arrhythmias (5.6%), cerebral palsy (5.6%), thyroid disorders (5.6%), stroke (5.6%)

Table S9: Condition percentages of the trajectories included in clusters for females, stratified by age. Each cluster presents the most frequent conditions including in the trajectories alongside the count of trajectories (N traj) and total patient numbers (N patients). Percentages (%) are calculated based on the total number of trajectories in each cluster. Clusters are presented in descending order of patient count within each age category.

N traj	N patients	Condition percentages and clusters
**a. Females $<$ 45 years**		
88	1713	mental illness (43.2%), reflux disorders (38.6%), chr. pain conditions (34.1%), chr. airway diseases (30.7%), insomnia (25.0%), chr. arthritis (21.6%), neuropathic pain (20.5%), IBD (18.2%), epilepsy (17.0%), diabetes (15.9%), dysphagia (8.0%), anaemia (6.8%), chr. diarrhoea (5.7%)
**b. Females $\geq$ 45 years**		
256	6101	cardiac arrhythmias (30.5%), CKD (26.6%), diabetes (24.2%), hypertension (23.4%), chr. arthritis (22.7%), CHD (22.7%), stroke (21.1%), heart failure (19.9%), anaemia (19.9%), chr. airway diseases (14.5%), epilepsy (13.3%), dementia (9.4%), mental illness (9.4%), cancer (6.6%), chr. pneumonia (6.3%), PVD (5.9%), chr. pain conditions (5.1%), dysphagia (5.1%)
183	4057	reflux disorders (48.1%), chr. arthritis (41.5%), neuropathic pain (23.5%), menopausal & perimenopausal (20.8%), chr. pain conditions (20.2%), insomnia (20.2%), chr. airway diseases (19.1%), mental illness (18.6%), anaemia (12.0%), dysphagia (8.7%), IBD (7.7%), hypertension (7.1%), chr. constipation (6.6%), chr. diarrhoea (6.6%)

Table S10: The shared LTC trajectories of length three identified within the male sub-population below 45 years of age. The column N Patients indicates the total number of patients that share this trajectory following this chronological order among the conditions.

Condition 1	Condition 2	Condition 3	Cluster	N Patients	Mortality %	Long Hospital Stay %
mental illness	insomnia	reflux disorders	1	41	14.6	29.3
epilepsy	mental illness	insomnia	1	30	18	50
mental illness	insomnia	chronic arthritis	1	23	20	34.8
chronic airway diseases	insomnia	reflux disorders	1	23	0	26.1
epilepsy	mental illness	reflux disorders	1	21	0	47.6
epilepsy	cerebral palsy	dysphagia	1	18	27	72
chronic airway diseases	insomnia	chronic arthritis	1	18	0	38.9
mental illness	chronic pain conditions	reflux disorders	1	18	0	44.4
chronic airway diseases	insomnia	neuropathic pain	1	16	0	31.2
reflux disorders	insomnia	neuropathic pain	1	15	0	33.3
mental illness	chronic pain conditions	chronic airway diseases	1	14	35	50
mental illness	reflux disorders	ibd	1	14	0	64.3
reflux disorders	chronic pain conditions	neuropathic pain	1	14	0	42.9
epilepsy	cerebral palsy	anaemia	1	14	35.7	65
mental illness	chronic pain conditions	insomnia	1	13	0	53.8
cerebral palsy	dysphagia	reflux disorders	1	13	36	65
epilepsy	chron constipation	dysphagia	1	13	0	62
reflux disorders	chronic pain conditions	insomnia	1	13	0	38.5
diabetes	hypertension	ckd	1	13	46.2	62
chronic airway diseases	chronic pain conditions	reflux disorders	1	13	0	38.5
mental illness	reflux disorders	anaemia	1	13	35	60
chronic airway diseases	chronic pain conditions	neuropathic pain	1	13	0	38.5
mental illness	insomnia	neuropathic pain	1	13	0	53.8
reflux disorders	insomnia	chronic arthritis	1	12	0	41.7
chronic airway diseases	chronic pain conditions	insomnia	1	12	0	50
epilepsy	hypertension	ckd	1	12	0	62
cerebral palsy	epilepsy	chron constipation	1	12	0	58.3
cerebral palsy	epilepsy	mental illness	1	12	0	41.7
chronic pain conditions	insomnia	neuropathic pain	1	11	42	45.5
epilepsy	mental illness	chronic pain conditions	1	11	0	54.5
epilepsy	hypertension	diabetes	1	11	0	54.5
chronic pain conditions	insomnia	chronic arthritis	1	10	0	50
chronic pain conditions	neuropathic pain	chronic arthritis	1	10	0	0
mental illness	chronic pain conditions	chronic arthritis	1	10	0	52
chronic airway diseases	chronic pain conditions	chronic arthritis	1	10	0	50
mental illness	reflux disorders	dysphagia	1	10	0	0
chronic arthritis	hypertension	diabetes	1	10	0	0

Table S11: The shared LTC trajectories of length three identified within the male sub-population aged 45 years and older. The column N Patients indicates the total number of patients that share this trajectory following this chronological order among the conditions.

Condition 1	Condition 2	Condition 3	Cluster	N Patients	Mortality %	Long Hospital Stay %
hypertension	diabetes	ckd	1	77	61	77.9
hypertension	ckd	cardiac arrhythmias	1	55	70.9	87.3
hypertension	cardiac arrhythmias	heart failure	1	54	63	87
hypertension	ckd	heart failure	1	53	75.5	92.5
diabetes	ckd	anaemia	1	53	62.3	86.8
hypertension	ckd	anaemia	1	50	72	88
chronic airway diseases	cardiac arrhythmias	heart failure	1	49	63.3	91.8
diabetes	ckd	cardiac arrhythmias	1	47	66	91.5
diabetes	cardiac arrhythmias	heart failure	1	46	76.1	89.1
chronic airway diseases	coronary heart disease	ckd	1	45	66.7	100
diabetes	ckd	heart failure	1	44	72.7	93.2
hypertension	coronary heart disease	ckd	1	43	60.5	90.7
coronary heart disease	cardiac arrhythmias	heart failure	1	43	69.8	90.7
chronic airway diseases	coronary heart disease	heart failure	1	42	66.7	92.9
coronary heart disease	cardiac arrhythmias	ckd	1	41	75.6	92.7
chronic airway diseases	cardiac arrhythmias	ckd	1	41	65.9	95.1
hypertension	diabetes	cardiac arrhythmias	1	41	61	85.4
hypertension	chronic arthritis	cardiac arrhythmias	1	41	56.1	85.4
chronic airway diseases	coronary heart disease	cardiac arrhythmias	1	40	50	97.5
chronic arthritis	cardiac arrhythmias	ckd	1	40	70	87.5
diabetes	coronary heart disease	ckd	1	37	54.1	94.6
reflux disorders	anaemia	ckd	1	37	70.3	86.5
chronic arthritis	cardiac arrhythmias	heart failure	1	36	66.7	80.6
ckd	cardiac arrhythmias	heart failure	1	35	74.3	94.3
chronic airway diseases	coronary heart disease	anaemia	1	35	51.4	88.6
hypertension	coronary heart disease	cardiac arrhythmias	1	35	60	97.1
hypertension	coronary heart disease	heart failure	1	35	71.4	94.3
reflux disorders	coronary heart disease	ckd	1	34	70.6	97.1
chronic arthritis	coronary heart disease	ckd	1	33	63.6	90.9
coronary heart disease	heart failure	ckd	1	33	75.8	93.9
hypertension	diabetes	heart failure	1	33	75.8	90.9
chronic airway diseases	heart failure	ckd	1	31	74.2	96.8
hypertension	cardiac arrhythmias	anaemia	1	31	48.4	80.6
epilepsy	diabetes	ckd	1	31	64.5	90.3
ckd	anaemia	heart failure	1	30	86.7	93.3
chronic airway diseases	cardiac arrhythmias	anaemia	1	30	46.7	83.3
diabetes	coronary heart disease	heart failure	1	29	69	96.6
reflux disorders	coronary heart disease	heart failure	1	28	67.9	92.9
coronary heart disease	chronic arthritis	cardiac arrhythmias	1	28	57.1	92.9
hypertension	coronary heart disease	reflux disorders	1	28	39.3	67.9
diabetes	ckd	pvd	1	27	55.6	88.9

Continued on next page

Table S11 – continued from previous page

Condition 1	Condition 2	Condition 3	Cluster	N Patients	Mortality %	Long Hospital Stay %
hypertension	chronic arthritis	heart failure	1	27	63	81.5
chronic pneumonia	cardiac arrhythmias	ckd	1	27	74.1	100
hypertension	coronary heart disease	anaemia	1	27	51.9	92.6
diabetes	coronary heart disease	cardiac arrhythmias	1	26	42.3	92.3
chronic arthritis	coronary heart disease	heart failure	1	26	76.9	92.3
chronic airway diseases	chronic pneumonia	ckd	1	26	80.8	100
coronary heart disease	anaemia	ckd	1	26	69.2	100
chronic airway diseases	chronic pneumonia	cardiac arrhythmias	1	25	72	100
chronic arthritis	heart failure	ckd	1	25	72	88
chronic airway diseases	coronary heart disease	diabetes	1	24	50	95.8
hypertension	coronary heart disease	chronic arthritis	1	24	45.8	83.3
anaemia	cardiac arrhythmias	heart failure	1	23	56.5	91.3
hypertension	diabetes	coronary heart disease	1	23	65.2	91.3
coronary heart disease	heart failure	anaemia	1	23	73.9	91.3
chronic arthritis	coronary heart disease	anaemia	1	23	65.2	87
hypertension	heart failure	anaemia	1	22	63.6	86.4
diabetes	coronary heart disease	anaemia	1	22	45.5	90.9
diabetes	cardiac arrhythmias	anaemia	1	22	50	81.8
anaemia	ckd	cardiac arrhythmias	1	22	77.3	100
hypertension	ckd	pvd	1	22	63.6	90.9
chronic airway diseases	heart failure	anaemia	1	21	66.7	85.7
reflux disorders	coronary heart disease	cardiac arrhythmias	1	20	55	95
hypertension	diabetes	pvd	1	20	50	65
diabetes	coronary heart disease	reflux disorders	1	19	57.9	89.5
chronic airway diseases	coronary heart disease	stroke	1	19	52.6	94.7
cancer	osteoporosis	anaemia	1	19	84.2	89.5
diabetes	heart failure	anaemia	1	19	63.2	84.2
coronary heart disease	pvd	ckd	1	18	55.6	94.4
cancer	ckd	anaemia	1	18	83.3	88.9
chronic airway diseases	coronary heart disease	pvd	1	18	55.6	100
epilepsy	diabetes	cardiac arrhythmias	1	17	52.9	82.4
hypertension	coronary heart disease	neuropathic pain	1	17	27	52.9
coronary heart disease	cardiac arrhythmias	anaemia	1	17	76.5	94.1
coronary heart disease	anaemia	cancer	1	16	68.8	87.5
diabetes	cardiac arrhythmias	pvd	1	16	62.5	75
stroke	coronary heart disease	ckd	1	15	86.7	100
hypertension	coronary heart disease	stroke	1	15	46.7	86.7
chronic diarrhoea	ckd	anaemia	1	15	86.7	100
coronary heart disease	pvd	cardiac arrhythmias	1	15	53.3	100
epilepsy	stroke	coronary heart disease	1	15	46.7	93.3
diabetes	coronary heart disease	pvd	1	15	60	93.3
chronic airway diseases	cardiac arrhythmias	pvd	1	14	64.3	92.9
chronic airway diseases	heart failure	pvd	1	14	71.4	100
cardiac arrhythmias	heart failure	pvd	1	14	85.7	100

Continued on next page

Table S11 – continued from previous page

Condition 1	Condition 2	Condition 3	Cluster	N Patients	Mortality %	Long Hospital Stay %
hypertension	coronary heart disease	pvd	1	14	64.3	85.7
chronic airway diseases	chronic pneumonia	heart failure	1	14	92.9	92.9
neuropathic pain	coronary heart disease	ckd	1	13	61.5	84.6
hypertension	cardiac arrhythmias	chronic pneumonia	1	13	61.5	84.6
chronic arthritis	cardiac arrhythmias	pvd	1	13	61.5	100
cardiac arrhythmias	ckd	pvd	1	13	100	92.3
diabetes	pvd	heart failure	1	12	83.3	91.7
cancer	ckd	heart failure	1	12	75	91.7
chronic arthritis	coronary heart disease	pvd	1	12	66.7	91.7
chronic pneumonia	cardiac arrhythmias	heart failure	1	12	75	100
chronic pneumonia	heart failure	ckd	1	12	83.3	100
coronary heart disease	heart failure	pvd	1	12	66.7	100
cardiac arrhythmias	anaemia	cancer	1	11	72.7	100
epilepsy	diabetes	heart failure	1	11	90.9	81.8
diabetes	coronary heart disease	stroke	1	11	90.9	100
diabetes	coronary heart disease	chronic arthritis	1	11	45.5	90.9
chronic diarrhoea	ckd	cardiac arrhythmias	1	11	63.6	90.9
chronic arthritis	coronary heart disease	stroke	1	11	72.7	90.9
heart failure	ckd	pvd	1	11	72.7	100
stroke	coronary heart disease	pvd	1	11	81.8	90.9
hypertension	cardiac arrhythmias	pvd	1	11	63.6	81.8
reflux disorders	coronary heart disease	stroke	1	11	54.5	90.9
diabetes	coronary heart disease	neuropathic pain	1	10	50	70
cancer	ckd	cardiac arrhythmias	1	10	100	80
chronic arthritis	heart failure	pvd	1	10	80	100
hypertension	ckd	parkinsons	1	10	100	100
hypertension	heart failure	pvd	1	10	70	90
chronic airway diseases	chronic arthritis	reflux disorders	2	42	33.3	57.1
hypertension	chronic arthritis	reflux disorders	2	38	44.7	57.9
mental illness	reflux disorders	anaemia	2	37	51.4	75.7
chronic airway diseases	chronic arthritis	cardiac arrhythmias	2	36	50	88.9
chronic airway diseases	reflux disorders	anaemia	2	36	47.2	80.6
chronic airway diseases	reflux disorders	mental illness	2	34	41.2	64.7
chronic airway diseases	reflux disorders	coronary heart disease	2	34	52.9	79.4
reflux disorders	chronic arthritis	cardiac arrhythmias	2	32	53.1	81.2
chronic airway diseases	reflux disorders	dysphagia	2	30	53.3	90
chronic arthritis	reflux disorders	anaemia	2	30	66.7	86.7
mental illness	insomnia	reflux disorders	2	28	35.7	71.4
chronic airway diseases	chronic arthritis	coronary heart disease	2	27	48.1	85.2
reflux disorders	coronary heart disease	anaemia	2	27	48.1	88.9
chronic arthritis	cardiac arrhythmias	anaemia	2	26	53.8	84.6
reflux disorders	chronic arthritis	coronary heart disease	2	26	50	80.8
mental illness	reflux disorders	chronic arthritis	2	25	28	64
chronic airway diseases	reflux disorders	neuropathic pain	2	25	40	60

Continued on next page

Table S11 – continued from previous page

Condition 1	Condition 2	Condition 3	Cluster	N Patients	Mortality %	Long Hospital Stay %
chronic airway diseases	reflux disorders	insomnia	2	25	24	64
chronic airway diseases	chronic arthritis	heart failure	2	24	66.7	87.5
hypertension	chronic arthritis	hearing loss	2	24	41.7	54.2
chronic airway diseases	chronic pain conditions	chronic arthritis	2	23	30.4	52.2
chronic airway diseases	chronic arthritis	insomnia	2	23	39.1	69.6
chronic airway diseases	chronic pain conditions	reflux disorders	2	22	27.3	50
chronic pain conditions	reflux disorders	mental illness	2	22	27.3	54.5
insomnia	reflux disorders	chronic arthritis	2	22	40.9	77.3
chronic airway diseases	chronic arthritis	neuropathic pain	2	22	36.4	50
chronic airway diseases	chronic arthritis	hearing loss	2	22	54.5	59.1
chronic arthritis	hearing loss	dementia	2	21	81	76.2
insomnia	chronic arthritis	cardiac arrhythmias	2	21	52.4	85.7
chronic arthritis	heart failure	anaemia	2	20	60	85
chronic pain conditions	reflux disorders	chronic arthritis	2	20	0	55
reflux disorders	anaemia	heart failure	2	20	80	95
mental illness	insomnia	chronic arthritis	2	20	30	75
chronic arthritis	reflux disorders	dysphagia	2	19	63.2	73.7
insomnia	reflux disorders	dysphagia	2	19	47.4	68.4
mental illness	reflux disorders	coronary heart disease	2	19	63.2	89.5
chronic pain conditions	neuropathic pain	chronic arthritis	2	19	0	42.1
mental illness	reflux disorders	neuropathic pain	2	19	36.8	73.7
chronic pain conditions	chronic arthritis	coronary heart disease	2	18	44.4	72.2
reflux disorders	neuropathic pain	chronic arthritis	2	18	27.8	66.7
chronic pain conditions	neuropathic pain	reflux disorders	2	18	0	0
reflux disorders	anaemia	cardiac arrhythmias	2	18	77.8	88.9
reflux disorders	chronic arthritis	heart failure	2	17	64.7	88.2
insomnia	reflux disorders	neuropathic pain	2	17	35.3	64.7
hearing loss	chronic arthritis	cardiac arrhythmias	2	17	52.9	88.2
hypertension	chronic arthritis	insomnia	2	17	47.1	64.7
chronic pain conditions	reflux disorders	insomnia	2	16	0	50
chronic pain conditions	chronic arthritis	cardiac arrhythmias	2	16	0	75
chronic airway diseases	coronary heart disease	neuropathic pain	2	16	56.2	93.8
reflux disorders	anaemia	cancer	2	16	68.8	81.2
insomnia	reflux disorders	anaemia	2	16	68.8	93.8
insomnia	reflux disorders	coronary heart disease	2	15	60	93.3
chronic airway diseases	chronic pain conditions	neuropathic pain	2	15	0	46.7
reflux disorders	anaemia	osteoporosis	2	15	66.7	80
hearing loss	chronic arthritis	reflux disorders	2	15	60	60
chronic airway diseases	reflux disorders	chronic constipation	2	15	40	73.3
coronary heart disease	reflux disorders	dysphagia	2	14	64.3	92.9
hypertension	chronic arthritis	neuropathic pain	2	14	0	42.9
coronary heart disease	reflux disorders	neuropathic pain	2	14	50	71.4
chronic airway diseases	neuropathic pain	insomnia	2	13	38.5	84.6
chronic pain conditions	neuropathic pain	insomnia	2	13	0	46.2

Continued on next page

Table S11 – continued from previous page

Condition 1	Condition 2	Condition 3	Cluster	N Patients	Mortality %	Long Hospital Stay %
chronic pain conditions	chronic arthritis	heart failure	2	12	66.7	91.7
insomnia	chronic arthritis	hearing loss	2	12	41.7	75
chronic pain conditions	chronic arthritis	insomnia	2	12	0	75
neuropathic pain	reflux disorders	anaemia	2	12	58.3	83.3
neuropathic pain	chronic arthritis	cardiac arrhythmias	2	12	50	75
mental illness	insomnia	neuropathic pain	2	11	0	63.6
chronic pain conditions	chronic airway diseases	heart failure	2	11	50	81.8
insomnia	neuropathic pain	chronic arthritis	2	11	0	45.5
chronic constipation	reflux disorders	chronic arthritis	2	11	63.6	72.7
chronic pain conditions	chronic airway diseases	insomnia	2	11	0	63.6
chronic pain conditions	neuropathic pain	pvd	2	10	0	70
hearing loss	chronic arthritis	heart failure	2	10	80	90
coronary heart disease	chronic arthritis	insomnia	2	10	50	90
mental illness	insomnia	chronic pain conditions	2	10	50	60
coronary heart disease	neuropathic pain	insomnia	2	10	50	80
neuropathic pain	chronic arthritis	coronary heart disease	2	10	50	70
insomnia	reflux disorders	chronic constipation	2	10	70	80
chronic pain conditions	reflux disorders	anaemia	2	10	50	70
insomnia	chronic arthritis	heart failure	2	10	50	90
chronic pain conditions	reflux disorders	coronary heart disease	2	10	50	60
epilepsy	chronic pneumonia	ckd	3	38	84.2	100
epilepsy	chronic pneumonia	dysphagia	3	35	80	100
hearing loss	dementia	epilepsy	3	28	85.7	57.1
epilepsy	chronic pneumonia	cardiac arrhythmias	3	26	84.6	100
mental illness	reflux disorders	dysphagia	3	25	60	68
mental illness	chronic constipation	anaemia	3	24	58.3	75
chronic airway diseases	chronic pneumonia	dysphagia	3	24	79.2	95.8
mental illness	chronic constipation	dysphagia	3	23	65.2	73.9
epilepsy	stroke	dysphagia	3	23	47.8	87
epilepsy	dementia	dysphagia	3	22	81.8	77.3
epilepsy	cerebral palsy	dysphagia	3	22	59.1	95.5
chronic constipation	anaemia	ckd	3	22	72.7	100
mental illness	chronic constipation	reflux disorders	3	22	59.1	86.4
thyroid disorders	dementia	epilepsy	3	20	85	65
epilepsy	chronic pneumonia	heart failure	3	18	94.4	100
chronic constipation	chronic diarrhoea	ckd	3	18	72.2	83.3
chronic constipation	reflux disorders	anaemia	3	17	52.9	82.4
reflux disorders	chronic constipation	dysphagia	3	16	75	75
hearing loss	dementia	dysphagia	3	16	81.2	87.5
chronic constipation	chronic pneumonia	ckd	3	15	66.7	100
mental illness	parkinsons	ckd	3	13	61.5	92.3
mental illness	chronic constipation	chronic pneumonia	3	13	69.2	92.3
thyroid disorders	dementia	dysphagia	3	13	92.3	76.9
coronary heart disease	stroke	dysphagia	3	12	83.3	100

Continued on next page

Table S11 – continued from previous page

Condition 1	Condition 2	Condition 3	Cluster	N Patients	Mortality %	Long Hospital Stay %
chronic constipation	cerebral palsy	dysphagia	3	12	75	100
epilepsy	chronic pneumonia	dementia	3	12	100	100
chronic constipation	chronic pneumonia	dysphagia	3	11	72.7	90.9
reflux disorders	dysphagia	dementia	3	11	54.5	63.6
chronic constipation	chronic diarrhoea	ibd	3	11	100	81.8
dementia	chronic pneumonia	dysphagia	3	11	90.9	100
cardiac arrhythmias	chronic pneumonia	dysphagia	3	10	80	100
chronic constipation	chronic diarrhoea	dysphagia	3	10	90	80
chronic airway diseases	chronic pneumonia	dementia	3	10	100	100
hypertension	dementia	dysphagia	3	10	100	80
mental illness	chronic constipation	chronic diarrhoea	3	10	80	70
chronic constipation	chronic pneumonia	heart failure	3	10	70	100

Table S12: The shared LTC trajectories of length three identified within the female sub-population aged less than 45 years. The column N Patients indicates the total number of patients that share this trajectory following this chronological order among the conditions.

Condition 1	Condition 2	Condition 3	Cluster	N Patients	Mortality %	Long Hospital Stay %
chronic airway diseases	mental illness	reflux disorders	1	55	0	27.3
mental illness	chronic pain conditions	reflux disorders	1	47	10.6	38.3
chronic airway diseases	chronic pain conditions	reflux disorders	1	46	13	39.1
chronic airway diseases	mental illness	chronic pain conditions	1	37	0	35.1
chronic airway diseases	mental illness	insomnia	1	36	14.1	50
epilepsy	mental illness	reflux disorders	1	34	0	38.2
mental illness	insomnia	chronic arthritis	1	31	0	38.7
chronic airway diseases	chronic pain conditions	chronic arthritis	1	30	0	46.7
mental illness	reflux disorders	insomnia	1	30	0	40
mental illness	chronic pain conditions	chronic arthritis	1	29	0	48.3
mental illness	chronic pain conditions	insomnia	1	29	0	31
chronic airway diseases	chronic pain conditions	neuropathic pain	1	28	0	46.4
chronic airway diseases	mental illness	diabetes	1	27	0	48.1
chronic airway diseases	chronic pain conditions	insomnia	1	27	0	33.3
mental illness	reflux disorders	chronic arthritis	1	26	0	42.3
chronic airway diseases	reflux disorders	insomnia	1	26	18.4	38.5
chronic airway diseases	mental illness	neuropathic pain	1	25	0	40
chronic airway diseases	reflux disorders	neuropathic pain	1	25	0	48
chronic airway diseases	reflux disorders	anaemia	1	25	0	44
chronic airway diseases	mental illness	chronic arthritis	1	25	19	28
epilepsy	cerebral palsy	dysphagia	1	24	33.3	79.2
mental illness	chronic pain conditions	neuropathic pain	1	24	0	25
mental illness	reflux disorders	neuropathic pain	1	23	20	34.8
chronic pain conditions	reflux disorders	insomnia	1	23	0	43.5
chronic pain conditions	reflux disorders	neuropathic pain	1	23	0	39.1
chronic airway diseases	chronic arthritis	reflux disorders	1	23	0	43.5
mental illness	reflux disorders	anaemia	1	23	0	47.8
mental illness	insomnia	diabetes	1	22	0	50
chronic airway diseases	mental illness	ibd	1	22	0	50
mental illness	ibd	insomnia	1	21	0	42.9
mental illness	reflux disorders	ibd	1	21	0	47.6
reflux disorders	insomnia	chronic arthritis	1	20	0	45
epilepsy	mental illness	chronic arthritis	1	20	0	45
chronic airway diseases	insomnia	chronic arthritis	1	19	0	25
mental illness	chronic diarrhoea	ibd	1	19	25.1	47.4
chronic airway diseases	reflux disorders	dysphagia	1	19	0	57.9
epilepsy	mental illness	insomnia	1	19	0	42.1
chronic airway diseases	reflux disorders	ibd	1	19	0	47.4
epilepsy	mental illness	ibd	1	18	0	61.1
mental illness	reflux disorders	dysphagia	1	18	0	44.4
mental illness	neuropathic pain	chronic arthritis	1	18	0	44.4

Continued on next page

Table S12 – continued from previous page

Condition 1	Condition 2	Condition 3	Cluster	N Patients	Mortality %	Long Hospital Stay %
mental illness	ibd	neuropathic pain	1	18	0	38.9
chronic airway diseases	neuropathic pain	chronic arthritis	1	18	0	38.9
chronic pain conditions	neuropathic pain	chronic arthritis	1	18	0	55.6
chronic airway diseases	insomnia	diabetes	1	17	0	41.2
mental illness	chronic pain conditions	ibd	1	17	0	35.3
epilepsy	mental illness	neuropathic pain	1	17	0	41.2
chronic pain conditions	reflux disorders	dysphagia	1	17	0	41.2
epilepsy	chronic pain conditions	reflux disorders	1	16	0	37.5
chronic airway diseases	diabetes	ckd	1	16	0	56.2
reflux disorders	ibd	neuropathic pain	1	16	0	50
chronic pain conditions	chronic arthritis	reflux disorders	1	16	0	62.5
chronic pain conditions	mental illness	diabetes	1	16	0	50
reflux disorders	insomnia	diabetes	1	16	0	62.5
chronic pain conditions	reflux disorders	anaemia	1	15	0	46.7
epilepsy	mental illness	chronic pain conditions	1	15	0	40
epilepsy	dysphagia	reflux disorders	1	15	40	53.3
mental illness	diabetes	chronic arthritis	1	15	0	53.3
mental illness	diabetes	hypertension	1	15	0	40
mental illness	diabetes	ckd	1	14	0	64.3
chronic pain conditions	ibd	insomnia	1	14	0	57.1
chronic pain conditions	insomnia	chronic arthritis	1	14	0	42.9
chronic pain conditions	ibd	reflux disorders	1	14	0	64.3
chronic pain conditions	ibd	neuropathic pain	1	14	0	71.4
chronic airway diseases	diabetes	chronic arthritis	1	14	0	57.1
chronic airway diseases	diabetes	hypertension	1	14	0	35.7
epilepsy	chronic constipation	dysphagia	1	14	50	50
chronic airway diseases	chronic pain conditions	ibd	1	13	0	69.2
epilepsy	chronic pain conditions	chronic arthritis	1	13	0	53.8
epilepsy	mental illness	diabetes	1	12	0	41.7
epilepsy	chronic pain conditions	insomnia	1	12	0	0
chronic airway diseases	neuropathic pain	hypertension	1	12	0	38
chronic pain conditions	neuropathic pain	hypertension	1	12	0	50
chronic pain conditions	insomnia	diabetes	1	11	0	43
chronic constipation	dysphagia	reflux disorders	1	11	42	45.5
epilepsy	chronic pain conditions	neuropathic pain	1	11	0	43
reflux disorders	chronic diarrhoea	ibd	1	11	0	45.5
ibd	reflux disorders	insomnia	1	11	0	45.5
mental illness	chronic diarrhoea	insomnia	1	11	0	54.5
mental illness	chronic diarrhoea	reflux disorders	1	11	0	72.7
chronic airway diseases	insomnia	ibd	1	11	0	63.6
epilepsy	chronic constipation	cerebral palsy	1	10	50	60
chronic airway diseases	mental illness	chronic diarrhoea	1	10	0	70
anaemia	reflux disorders	chronic arthritis	1	10	0	53
reflux disorders	anaemia	ckd	1	10	0	53

Continued on next page

Table S12 – continued from previous page

Condition 1	Condition 2	Condition 3	Cluster	N Patients	Mortality %	Long Hospital Stay %
insomnia	chronic pain conditions	neuropathic pain	1	10	0	0
diabetes	ckd	anaemia	1	10	50	80
neuropathic pain	chronic arthritis	reflux disorders	1	10	0	50

Table S13: The shared LTC trajectories of length three identified within the female sub-population aged 45 years and older. The column N Patients indicates the total number of patients that share this trajectory following this chronological order among the conditions

Condition 1	Condition 2	Condition 3	Cluster	N Patients	Mortality %	Long Hospital Stay %
hypertension	chronic arthritis	ckd	1	77	54.5	74.0
chronic airway diseases	chronic arthritis	ckd	1	63	50.8	65.1
diabetes	hypertension	ckd	1	62	67.7	77.4
hypertension	ckd	anaemia	1	62	66.1	74.2
hypertension	ckd	cardiac arrhythmias	1	61	68.9	82.0
hypertension	chronic arthritis	cardiac arrhythmias	1	59	57.6	81.4
hypertension	ckd	heart failure	1	59	84.7	86.4
chronic airway diseases	diabetes	ckd	1	57	59.6	66.7
hypertension	cardiac arrhythmias	heart failure	1	56	71.4	89.3
diabetes	anaemia	ckd	1	54	68.5	85.2
mental illness	diabetes	ckd	1	54	55.6	75.9
hypertension	ckd	dementia	1	54	74.1	83.3
chronic arthritis	anaemia	ckd	1	53	62.3	88.7
mental illness	chronic arthritis	ckd	1	51	54.9	74.5
chronic arthritis	cardiac arrhythmias	ckd	1	50	74.0	84.0
hypertension	chronic arthritis	heart failure	1	50	66.0	80.0
chronic arthritis	cardiac arrhythmias	heart failure	1	49	65.3	83.7
epilepsy	hypertension	ckd	1	46	58.7	71.7
chronic arthritis	ckd	dementia	1	46	78.3	90.0
chronic airway diseases	chd	heart failure	1	44	65.9	81.8
hypertension	diabetes	anaemia	1	42	57.1	66.7
chronic arthritis	chd	heart failure	1	42	61.9	83.3
chronic arthritis	ckd	heart failure	1	41	78.0	85.4
mental illness	chronic airway diseases	diabetes	1	41	36.6	70.7
hypertension	chronic arthritis	anaemia	1	41	63.4	85.4
hypertension	chd	heart failure	1	41	78.0	89.0
diabetes	ckd	cardiac arrhythmias	1	40	72.5	82.5
chronic airway diseases	diabetes	anaemia	1	40	55.0	77.5
diabetes	ckd	dementia	1	40	82.5	88.0
epilepsy	chronic arthritis	ckd	1	39	59.0	84.6
diabetes	anaemia	cardiac arrhythmias	1	39	74.4	88.0
chronic airway diseases	chronic arthritis	cardiac arrhythmias	1	38	57.9	81.6
hypertension	ckd	stroke	1	38	55.3	86.8
diabetes	ckd	heart failure	1	38	86.8	84.2
hypertension	diabetes	cardiac arrhythmias	1	37	67.6	78.4
chronic airway diseases	cardiac arrhythmias	heart failure	1	37	64.9	87.0
hypertension	chronic arthritis	hearing loss	1	36	50.0	72.2
mental illness	chronic airway diseases	chd	1	36	52.8	77.8
hypertension	ckd	chd	1	36	58.3	83.3
hypertension	chronic arthritis	chd	1	35	65.7	82.9
mental illness	chronic airway diseases	cardiac arrhythmias	1	35	48.6	82.9
hypertension	stroke	dementia	1	35	68.6	86.0
hypertension	chd	cardiac arrhythmias	1	35	65.7	85.7
ckd	cardiac arrhythmias	heart failure	1	35	68.6	87.0
chronic airway diseases	chd	cardiac arrhythmias	1	34	64.7	79.4

Continued on next page

Table S13 – continued from previous page

Condition 1	Condition 2	Condition 3	Cluster	N Patients	Mortality %	Long Hospital Stay %
diabetes	hypertension	chronic arthritis	1	34	50.0	73.5
thyroid disorders	dementia	dysphagia	1	33	84.8	78.8
mental illness	diabetes	anaemia	1	33	60.6	75.8
chronic arthritis	anaemia	cardiac arrhythmias	1	33	69.7	85.0
mental illness	chronic airway diseases	heart failure	1	33	66.7	85.0
chronic airway diseases	diabetes	cardiac arrhythmias	1	33	51.5	75.8
chronic airway diseases	chd	ckd	1	33	72.7	81.8
mental illness	chronic arthritis	heart failure	1	32	53.1	81.2
chronic pain conditions	chronic arthritis	ckd	1	32	59.4	65.6
diabetes	stroke	ckd	1	32	81.2	85.0
chronic airway diseases	chronic arthritis	chd	1	32	40.6	75.0
mental illness	chronic arthritis	cardiac arrhythmias	1	31	64.5	80.6
hypertension	cardiac arrhythmias	stroke	1	31	80.6	83.0
chronic airway diseases	chd	anaemia	1	31	58.1	83.9
diabetes	chd	ckd	1	30	80.0	83.0
epilepsy	hypertension	chronic arthritis	1	30	33.3	56.7
chronic airway diseases	chronic arthritis	heart failure	1	30	46.7	80.0
chronic arthritis	chd	cardiac arrhythmias	1	30	70.0	83.0
diabetes	chd	chronic arthritis	1	30	63.3	83.0
chronic airway diseases	diabetes	hypertension	1	29	44.8	58.6
epilepsy	chronic arthritis	anaemia	1	28	39.3	83.0
epilepsy	chronic arthritis	cardiac arrhythmias	1	28	57.1	82.0
anaemia	ckd	heart failure	1	28	82.0	82.0
hypertension	chronic pain conditions	chronic arthritis	1	28	39.3	57.1
diabetes	cardiac arrhythmias	heart failure	1	28	67.9	82.0
chd	cardiac arrhythmias	ckd	1	28	82.0	83.0
chronic arthritis	chd	anaemia	1	27	59.3	83.0
diabetes	hypertension	stroke	1	27	74.1	82.0
chd	anaemia	ckd	1	27	66.7	82.0
diabetes	stroke	anaemia	1	27	77.8	82.0
cardiac arrhythmias	chd	heart failure	1	27	77.8	82.0
mental illness	diabetes	cardiac arrhythmias	1	27	63.0	81.5
diabetes	chd	heart failure	1	27	83.0	82.0
chronic arthritis	ckd	stroke	1	27	59.3	77.8
reflux disorders	chd	heart failure	1	26	69.2	76.9
chronic arthritis	chd	ckd	1	26	65.4	80.8
chronic airway diseases	cardiac arrhythmias	ckd	1	26	79.0	81.0
hypertension	chronic arthritis	osteoporosis	1	26	57.7	80.8
hypertension	diabetes	heart failure	1	26	69.2	76.9
cancer	anaemia	ckd	1	25	80.0	80.0
mental illness	chronic arthritis	chd	1	25	48.0	80.0
hypertension	heart failure	stroke	1	25	80.0	80.0
hypertension	cardiac arrhythmias	anaemia	1	25	76.0	80.0
chronic arthritis	anaemia	stroke	1	25	68.0	80.0
mental illness	diabetes	hypertension	1	25	40.0	64.0
diabetes	chd	anaemia	1	25	68.0	80.0
ckd	anaemia	cardiac arrhythmias	1	24	80.0	81.0
epilepsy	stroke	dysphagia	1	24	54.2	100.0
epilepsy	dementia	dysphagia	1	24	80.0	79.0

Continued on next page

Table S13 – continued from previous page

Condition 1	Condition 2	Condition 3	Cluster	N Patients	Mortality %	Long Hospital Stay %
chd	anaemia	cardiac arrhythmias	1	24	75.0	79.0
diabetes	stroke	dementia	1	24	79.2	81.0
chronic arthritis	cardiac arrhythmias	stroke	1	24	79.2	80.0
hypertension	stroke	anaemia	1	24	75.0	75.0
epilepsy	stroke	anaemia	1	23	52.2	80.0
chronic airway diseases	chd	diabetes	1	23	52.2	69.6
epilepsy	chronic arthritis	hearing loss	1	23	34.8	78.3
ckd	chd	heart failure	1	23	65.2	80.0
hypertension	diabetes	neuropathic pain	1	23	39.1	56.5
diabetes	anaemia	heart failure	1	23	80.0	80.0
epilepsy	chronic arthritis	osteoporosis	1	22	50.0	75.0
ckd	dementia	dysphagia	1	22	79.0	100.0
epilepsy	stroke	dementia	1	22	79.0	79.0
reflux disorders	chd	ckd	1	22	68.2	72.7
chronic airway diseases	chronic pain conditions	hypertension	1	22	22.7	31.8
epilepsy	stroke	ckd	1	22	77.3	100.0
reflux disorders	chd	cardiac arrhythmias	1	22	68.2	75.0
thyroid disorders	dementia	stroke	1	22	78.0	78.0
chronic airway diseases	diabetes	stroke	1	22	63.6	72.7
neuropathic pain	hypertension	ckd	1	22	63.6	63.6
chronic airway diseases	diabetes	heart failure	1	22	77.3	68.2
anaemia	ckd	dementia	1	22	79.0	78.0
cancer	osteoporosis	cardiac arrhythmias	1	22	63.6	79.0
chronic arthritis	anaemia	cancer	1	22	63.6	68.2
epilepsy	chronic pneumonia	dysphagia	1	22	68.2	100.0
chronic arthritis	heart failure	anaemia	1	22	72.7	75.0
anaemia	cardiac arrhythmias	heart failure	1	22	79.0	75.0
chronic pain conditions	hypertension	ckd	1	22	59.1	68.2
hypertension	chd	anaemia	1	22	77.3	75.0
chronic arthritis	pvd	cardiac arrhythmias	1	21	76.2	76.0
cardiac arrhythmias	ckd	dementia	1	21	76.0	78.0
hypertension	stroke	dysphagia	1	21	78.0	78.0
hypertension	chd	reflux disorders	1	21	61.9	78.0
chronic airway diseases	heart failure	anaemia	1	21	71.4	78.0
diabetes	stroke	dysphagia	1	21	76.2	100.0
chronic arthritis	osteoporosis	cardiac arrhythmias	1	21	66.7	78.0
epilepsy	hypertension	cardiac arrhythmias	1	21	66.7	75.0
hypertension	chd	stroke	1	20	75.0	76.0
chronic pain conditions	hypertension	diabetes	1	20	0.0	25.0
ibd	cardiac arrhythmias	heart failure	1	20	70.0	76.0
chronic arthritis	cardiac arrhythmias	cancer	1	20	75.0	75.0
neuropathic pain	diabetes	ckd	1	20	50.0	74.0
anaemia	ckd	stroke	1	20	65.0	75.0
diabetes	hypertension	chd	1	20	55.0	74.0
mental illness	diabetes	chd	1	20	55.0	75.0
mental illness	diabetes	heart failure	1	20	60.0	75.0
hypertension	chronic arthritis	pvd	1	20	60.0	70.0
diabetes	cardiac arrhythmias	stroke	1	20	75.0	76.0
chronic airway diseases	cardiac arrhythmias	stroke	1	20	75.0	75.0

Continued on next page

Table S13 – continued from previous page

Condition 1	Condition 2	Condition 3	Cluster	N Patients	Mortality %	Long Hospital Stay %
chronic pain conditions	chronic arthritis	cardiac arrhythmias	1	20	50.0	75.0
hypertension	cardiac arrhythmias	cancer	1	19	75.0	75.0
mental illness	diabetes	stroke	1	19	68.4	75.0
chronic pain conditions	chronic airway diseases	diabetes	1	19	42.1	52.6
cancer	cardiac arrhythmias	ckd	1	19	73.7	75.0
diabetes	chd	cardiac arrhythmias	1	19	75.0	75.0
epilepsy	hypertension	stroke	1	19	68.4	75.0
chronic airway diseases	chd	stroke	1	19	75.0	75.0
chronic airway diseases	cardiac arrhythmias	anaemia	1	19	68.4	100.0
ibd	anaemia	cardiac arrhythmias	1	19	68.4	75.0
anaemia	cardiac arrhythmias	stroke	1	18	72.2	72.0
stroke	ckd	dementia	1	18	73.0	72.0
cancer	anaemia	cardiac arrhythmias	1	18	73.0	100.0
chronic arthritis	heart failure	stroke	1	18	73.0	72.0
chronic airway diseases	chronic pneumonia	cardiac arrhythmias	1	18	73.0	72.0
diabetes	chd	stroke	1	18	72.0	72.0
epilepsy	diabetes	ckd	1	18	55.6	72.0
chd	heart failure	stroke	1	18	74.0	73.0
chd	heart failure	anaemia	1	18	61.1	72.0
mental illness	chronic pain conditions	hypertension	1	18	0.0	33.3
stroke	cardiac arrhythmias	ckd	1	18	74.0	73.0
ibd	cardiac arrhythmias	ckd	1	17	64.7	72.0
hearing loss	chronic arthritis	ckd	1	17	73.0	72.0
hearing loss	chronic arthritis	cardiac arrhythmias	1	17	73.0	72.0
epilepsy	chronic arthritis	heart failure	1	17	52.9	72.0
thyroid disorders	dementia	chronic pneumonia	1	17	100.0	72.0
epilepsy	diabetes	cardiac arrhythmias	1	17	52.9	72.0
chronic arthritis	pvd	ckd	1	17	73.0	72.0
epilepsy	hypertension	diabetes	1	17	29.4	47.1
chronic pain conditions	chronic arthritis	chd	1	17	47.1	64.7
chd	ckd	dementia	1	17	72.0	72.0
cardiac arrhythmias	heart failure	stroke	1	16	71.0	70.0
hypertension	heart failure	anaemia	1	16	70.0	69.0
reflux disorders	chd	diabetes	1	16	43.8	68.8
osteoporosis	cardiac arrhythmias	heart failure	1	16	62.5	69.0
chronic airway diseases	heart failure	stroke	1	16	70.0	69.0
ckd	heart failure	stroke	1	16	70.0	70.0
pvd	cardiac arrhythmias	ckd	1	16	100.0	71.0
diabetes	epilepsy	dementia	1	15	68.0	100.0
chronic airway diseases	cardiac arrhythmias	osteoporosis	1	15	69.0	100.0
diabetes	anaemia	cancer	1	15	66.7	65.0
epilepsy	chronic pneumonia	cardiac arrhythmias	1	15	69.0	100.0
epilepsy	dementia	chronic pneumonia	1	15	100.0	69.0
hypertension	cardiac arrhythmias	osteoporosis	1	15	66.7	69.0
chronic airway diseases	chronic pneumonia	ckd	1	15	69.0	100.0
chronic airway diseases	cardiac arrhythmias	cancer	1	15	69.0	100.0
neuropathic pain	hypertension	cardiac arrhythmias	1	14	57.1	57.1
diabetes	anaemia	ibd	1	14	50.0	62.0
reflux disorders	anaemia	stroke	1	14	64.3	65.0

Continued on next page

Table S13 – continued from previous page

Condition 1	Condition 2	Condition 3	Cluster	N Patients	Mortality %	Long Hospital Stay %
chronic arthritis	chd	stroke	1	14	63.0	65.0
diabetes	epilepsy	stroke	1	14	50.0	65.0
anaemia	heart failure	stroke	1	14	67.0	65.0
diabetes	epilepsy	chronic arthritis	1	14	64.3	100.0
diabetes	stroke	heart failure	1	14	65.0	65.0
chronic arthritis	ckd	chronic pneumonia	1	14	63.0	100.0
chronic airway diseases	chd	pvd	1	14	64.3	65.0
chronic pain conditions	mental illness	diabetes	1	14	0.0	42.9
epilepsy	stroke	cardiac arrhythmias	1	14	64.3	100.0
chd	stroke	anaemia	1	14	64.0	100.0
cancer	cardiac arrhythmias	chd	1	14	64.3	100.0
reflux disorders	chd	stroke	1	13	65.0	65.0
anaemia	cancer	osteoporosis	1	13	65.0	65.0
epilepsy	chronic pneumonia	ckd	1	13	64.0	100.0
chronic pain conditions	hypertension	cardiac arrhythmias	1	13	65.0	65.0
epilepsy	hypertension	chronic pain conditions	1	13	38.5	53.8
ibd	cardiac arrhythmias	cancer	1	13	65.0	65.0
chd	anaemia	cancer	1	13	65.0	65.0
chronic airway diseases	chronic pneumonia	heart failure	1	13	65.0	65.0
stroke	dysphagia	dementia	1	13	65.0	100.0
diabetes	ckd	chronic pneumonia	1	13	65.0	65.0
epilepsy	diabetes	anaemia	1	12	41.7	61.0
ckd	anaemia	ibd	1	12	62.0	61.0
epilepsy	hypertension	heart failure	1	12	62.0	62.0
chd	stroke	ckd	1	12	62.0	62.0
chd	stroke	dementia	1	12	62.0	100.0
cardiac arrhythmias	stroke	dementia	1	12	61.0	62.0
cardiac arrhythmias	chd	stroke	1	12	62.0	62.0
mental illness	diabetes	epilepsy	1	12	43.3	62.0
ckd	chronic pneumonia	cardiac arrhythmias	1	12	62.0	100.0
ckd	chronic pneumonia	dysphagia	1	12	62.0	62.0
anaemia	stroke	dementia	1	12	62.0	100.0
chronic airway diseases	chronic pneumonia	dementia	1	12	62.0	100.0
osteoporosis	cardiac arrhythmias	ckd	1	12	62.0	62.0
chronic arthritis	chd	pvd	1	12	58.3	62.0
anaemia	cardiac arrhythmias	osteoporosis	1	12	62.0	100.0
hypertension	cardiac arrhythmias	pvd	1	11	58.0	100.0
pvd	chd	heart failure	1	11	59.0	59.0
chronic pain conditions	hypertension	chd	1	11	54.5	55.0
hypertension	chd	pvd	1	11	59.0	55.0
chronic arthritis	pvd	heart failure	1	11	58.0	55.0
diabetes	epilepsy	dysphagia	1	11	59.0	100.0
ibd	anaemia	cancer	1	11	59.0	55.0
ibd	anaemia	heart failure	1	11	59.0	55.0
pvd	ckd	heart failure	1	11	58.0	55.0
dementia	chronic pneumonia	dysphagia	1	11	58.0	55.0
chd	chronic arthritis	hearing loss	1	11	54.5	55.0
epilepsy	hypertension	neuropathic pain	1	11	0.0	55.0
chronic airway diseases	cardiac arrhythmias	pvd	1	11	55.0	100.0

Continued on next page

Table S13 – continued from previous page

Condition 1	Condition 2	Condition 3	Cluster	N Patients	Mortality %	Long Hospital Stay %
cancer	cardiac arrhythmias	heart failure	1	10	50.0	52.0
chd	pvd	cardiac arrhythmias	1	10	53.0	52.0
chronic pneumonia	ckd	dementia	1	10	53.0	100.0
ibd	cardiac arrhythmias	stroke	1	10	52.0	100.0
ibd	cardiac arrhythmias	chd	1	10	50.0	52.0
pvd	cardiac arrhythmias	heart failure	1	10	52.0	52.0
dysphagia	stroke	ckd	1	10	54.0	100.0
chd	stroke	dysphagia	1	10	100.0	100.0
hypertension	ckd	chronic pneumonia	1	10	52.0	100.0
hypertension	stroke	pvd	1	10	52.0	52.0
epilepsy	diabetes	heart failure	1	10	52.0	52.0
diabetes	cardiac arrhythmias	cancer	1	10	52.0	52.0
cancer	anaemia	heart failure	1	10	52.0	100.0
hearing loss	chronic arthritis	heart failure	1	10	52.0	52.0
chronic airway diseases	diabetes	epilepsy	1	10	52.0	52.0
chronic airway diseases	reflux disorders	chronic arthritis	2	58	25.9	48.3
mental illness	chronic airway diseases	reflux disorders	2	50	26.0	54.0
mental illness	chronic airway diseases	chronic arthritis	2	50	32.0	56.0
mental illness	reflux disorders	chronic arthritis	2	50	34.0	52.0
chronic airway diseases	reflux disorders	anaemia	2	48	41.7	77.1
mental illness	chronic arthritis	anaemia	2	47	51.1	74.5
reflux disorders	chronic arthritis	ckd	2	47	48.9	76.6
mental illness	reflux disorders	anaemia	2	46	39.1	71.7
reflux disorders	anaemia	ckd	2	45	57.8	82.2
mental illness	chronic pain conditions	chronic arthritis	2	43	11.6	60.5
chronic arthritis	reflux disorders	anaemia	2	42	52.4	83.3
mental illness	reflux disorders	menopausal & perimenopausal	2	42	31.0	57.1
chronic airway diseases	reflux disorders	menopausal & perimenopausal	2	39	25.6	59.0
hypertension	chronic arthritis	reflux disorders	2	38	57.9	76.3
chronic airway diseases	chronic arthritis	osteoporosis	2	37	64.9	73.0
chronic airway diseases	chronic pain conditions	chronic arthritis	2	36	25.0	55.6
reflux disorders	menopausal & perimenopausal	chronic arthritis	2	36	16.7	55.6
mental illness	chronic pain conditions	reflux disorders	2	35	14.3	42.9
chronic arthritis	reflux disorders	dysphagia	2	35	65.7	74.3
chronic airway diseases	chronic arthritis	anaemia	2	34	58.8	73.5
chronic pain conditions	reflux disorders	chronic arthritis	2	34	20.6	29.4
mental illness	reflux disorders	dysphagia	2	34	38.2	50.0
reflux disorders	chronic arthritis	cardiac arrhythmias	2	33	45.5	75.8
chronic airway diseases	reflux disorders	dysphagia	2	33	48.5	48.5
mental illness	chronic arthritis	menopausal & perimenopausal	2	33	36.4	57.6
mental illness	reflux disorders	neuropathic pain	2	33	33.3	51.5
mental illness	chronic airway diseases	neuropathic pain	2	32	28.1	56.2
mental illness	chronic arthritis	neuropathic pain	2	32	34.4	59.4
mental illness	insomnia	chronic arthritis	2	32	21.9	56.2
chronic airway diseases	insomnia	chronic arthritis	2	32	34.4	59.4
mental illness	chronic pain conditions	neuropathic pain	2	32	17.0	46.9
chronic airway diseases	chronic pain conditions	reflux disorders	2	32	18.8	46.9
chronic airway diseases	chronic arthritis	neuropathic pain	2	32	28.1	68.8
hypertension	chronic arthritis	menopausal & perimenopausal	2	32	28.1	56.2

Continued on next page

Table S13 – continued from previous page

Condition 1	Condition 2	Condition 3	Cluster	N Patients	Mortality %	Long Hospital Stay %
chronic airway diseases	reflux disorders	neuropathic pain	2	31	25.8	64.5
hypertension	chronic arthritis	neuropathic pain	2	31	48.4	74.2
menopausal & perimenopausal	chronic arthritis	ckd	2	31	41.9	77.4
mental illness	chronic pain conditions	menopausal & perimenopausal	2	31	16.9	48.4
chronic pain conditions	reflux disorders	neuropathic pain	2	31	22.6	38.7
chronic airway diseases	chronic arthritis	hearing loss	2	31	48.4	64.5
reflux disorders	chronic arthritis	neuropathic pain	2	30	23.3	46.7
mental illness	chronic arthritis	osteoporosis	2	30	46.7	63.3
menopausal & perimenopausal	chronic arthritis	anaemia	2	30	50.0	76.7
mental illness	insomnia	reflux disorders	2	30	20.0	60.0
reflux disorders	chronic arthritis	osteoporosis	2	29	51.7	75.9
menopausal & perimenopausal	chronic arthritis	cardiac arrhythmias	2	28	60.7	78.6
mental illness	chronic airway diseases	insomnia	2	28	32.1	67.9
mental illness	chronic arthritis	hearing loss	2	28	50.0	67.9
menopausal & perimenopausal	reflux disorders	anaemia	2	27	66.7	77.8
reflux disorders	anaemia	cardiac arrhythmias	2	27	63.0	82.0
chronic pain conditions	neuropathic pain	chronic arthritis	2	27	18.5	40.7
reflux disorders	chronic arthritis	heart failure	2	27	59.3	82.0
reflux disorders	chronic arthritis	chd	2	26	50.0	80.8
chronic airway diseases	chronic arthritis	menopausal & perimenopausal	2	26	34.6	50.0
reflux disorders	mental illness	diabetes	2	26	26.9	53.8
chronic airway diseases	reflux disorders	ibd	2	26	38.5	61.5
chronic airway diseases	reflux disorders	insomnia	2	26	23.1	61.5
chronic airway diseases	diabetes	neuropathic pain	2	26	34.6	65.4
chronic airway diseases	mental illness	chronic pain conditions	2	26	23.1	46.2
hypertension	chronic arthritis	insomnia	2	25	68.0	80.0
reflux disorders	ibd	anaemia	2	25	40.0	76.0
chronic airway diseases	chronic pain conditions	menopausal & perimenopausal	2	25	24.0	44.0
reflux disorders	chronic arthritis	hearing loss	2	24	50.0	62.5
mental illness	diabetes	neuropathic pain	2	24	29.2	54.2
reflux disorders	chronic arthritis	insomnia	2	24	41.7	62.5
chronic arthritis	menopausal & perimenopausal	neuropathic pain	2	24	29.2	54.2
menopausal & perimenopausal	chronic arthritis	heart failure	2	24	41.7	80.0
epilepsy	chronic constipation	reflux disorders	2	24	62.5	79.2
chronic pain conditions	reflux disorders	menopausal & perimenopausal	2	24	20.0	41.7
neuropathic pain	chronic arthritis	ckd	2	24	50.0	70.8
epilepsy	cerebral palsy	dysphagia	2	23	65.2	75.0
chronic constipation	reflux disorders	mental illness	2	23	56.5	47.8
mental illness	insomnia	menopausal & perimenopausal	2	23	26.1	47.8
epilepsy	chronic constipation	dysphagia	2	23	65.2	73.9
chronic airway diseases	chd	reflux disorders	2	23	65.2	80.0
chronic pain conditions	menopausal & perimenopausal	chronic arthritis	2	23	21.7	39.1
epilepsy	chronic arthritis	menopausal & perimenopausal	2	22	36.4	68.2
menopausal & perimenopausal	reflux disorders	neuropathic pain	2	22	36.4	54.5
menopausal & perimenopausal	chronic arthritis	hearing loss	2	22	22.7	59.1
chronic pain conditions	menopausal & perimenopausal	neuropathic pain	2	22	22.7	45.5
chronic arthritis	neuropathic pain	diabetes	2	21	38.1	76.2
chronic pain conditions	neuropathic pain	hypertension	2	21	23.8	42.9
mental illness	insomnia	neuropathic pain	2	21	0.0	52.4

Continued on next page

Table S13 – continued from previous page

Condition 1	Condition 2	Condition 3	Cluster	N Patients	Mortality %	Long Hospital Stay %
reflux disorders	menopausal & perimenopausal	insomnia	2	21	47.6	57.1
chronic airway diseases	chronic pain conditions	neuropathic pain	2	21	0.0	52.4
chronic pain conditions	insomnia	chronic arthritis	2	21	0.0	42.9
mental illness	neuropathic pain	hypertension	2	20	30.0	50.0
chronic pain conditions	reflux disorders	anaemia	2	20	45.0	75.0
chronic pain conditions	insomnia	reflux disorders	2	20	25.0	60.0
chronic pain conditions	chronic arthritis	hearing loss	2	20	30.0	45.0
reflux disorders	chronic diarrhea	ibd	2	20	30.0	75.0
reflux disorders	anaemia	cancer	2	20	65.0	65.0
menopausal & perimenopausal	chronic arthritis	chd	2	20	40.0	74.0
menopausal & perimenopausal	chronic arthritis	osteoporosis	2	20	45.0	60.0
mental illness	reflux disorders	chd	2	20	45.0	75.0
insomnia	chronic arthritis	ckd	2	19	36.8	73.7
reflux disorders	chd	anaemia	2	19	52.6	73.7
chronic pain conditions	neuropathic pain	diabetes	2	19	0.0	42.1
reflux disorders	neuropathic pain	diabetes	2	19	26.3	68.4
epilepsy	chronic arthritis	reflux disorders	2	19	63.2	75.0
insomnia	menopausal and perimenopausal	chronic arthritis	2	19	26.3	47.4
hearing loss	chronic arthritis	anaemia	2	19	75.0	75.0
hypertension	chronic pain conditions	menopausal and perimenopausal	2	19	42.1	63.2
reflux disorders	insomnia	neuropathic pain	2	19	25.1	42.1
hypertension	chronic pain conditions	reflux disorders	2	18	38.9	55.6
chronic pain conditions	chronic arthritis	anaemia	2	18	55.6	70.0
reflux disorders	chronic constipation	dysphagia	2	18	61.1	50.0
chronic airway diseases	reflux disorders	chronic constipation	2	18	27.8	61.1
mental illness	reflux disorders	ibd	2	18	0.0	55.6
chronic airway diseases	insomnia	neuropathic pain	2	17	0.0	72.0
reflux disorders	ibd	cardiac arrhythmias	2	17	52.9	72.0
chronic airway diseases	chronic arthritis	pvd	2	17	64.7	72.0
chronic airway diseases	chronic pain conditions	insomnia	2	17	41.2	72.0
chronic pain conditions	reflux disorders	ibd	2	17	35.3	64.7
mental illness	chronic pain conditions	insomnia	2	17	29.4	72.0
chronic pain conditions	chronic arthritis	osteoporosis	2	17	52.9	70.6
chronic pain conditions	chronic arthritis	heart failure	2	17	58.8	72.0
chronic airway diseases	neuropathic pain	hypertension	2	17	41.2	35.3
chronic arthritis	reflux disorders	ibd	2	16	50.0	69.0
reflux disorders	dysphagia	dementia	2	16	56.2	69.0
chronic pain conditions	reflux disorders	dysphagia	2	16	37.5	43.8
insomnia	reflux disorders	dysphagia	2	16	31.2	50.0
chronic constipation	reflux disorders	chronic arthritis	2	16	37.5	62.5
chronic airway diseases	insomnia	menopausal and perimenopausal	2	16	37.5	68.8
hearing loss	chronic arthritis	osteoporosis	2	16	62.5	68.8
menopausal and perimenopausal	chronic pain conditions	insomnia	2	15	30.7	40.0
neuropathic pain	chronic arthritis	anaemia	2	15	46.7	66.7
insomnia	chronic arthritis	neuropathic pain	2	15	0.0	53.3
menopausal and perimenopausal	neuropathic pain	hypertension	2	15	33.3	30.0
reflux disorders	dysphagia	stroke	2	15	53.3	69.0
chronic pain conditions	reflux disorders	chronic constipation	2	15	40.0	53.3
chronic pain conditions	reflux disorders	chd	2	15	46.7	67.0

Continued on next page

Table S13 – continued from previous page

Condition 1	Condition 2	Condition 3	Cluster	N Patients	Mortality %	Long Hospital Stay %
chronic pain conditions	chronic airway diseases	cardiac arrhythmias	2	15	66.7	69.0
chronic airway diseases	chronic pneumonia	dysphagia	2	15	100.0	68.0
menopausal and perimenopausal	reflux disorders	dysphagia	2	15	60.0	66.7
reflux disorders	anaemia	heart failure	2	15	60.0	69.0
reflux disorders	neuropathic pain	hypertension	2	15	36.7	40.0
neuropathic pain	chronic arthritis	cardiac arrhythmias	2	14	50.0	65.0
chronic diarrhea	ibd	anaemia	2	14	57.1	65.0
chronic diarrhea	ibd	cardiac arrhythmias	2	14	64.3	65.0
ibd	reflux disorders	menopausal and perimenopausal	2	14	0.0	50.0
ibd	reflux disorders	dysphagia	2	14	50.0	64.3
insomnia	chronic arthritis	cardiac arrhythmias	2	14	35.7	65.0
chronic constipation	reflux disorders	menopausal and perimenopausal	2	13	46.2	53.8
chronic constipation	reflux disorders	anaemia	2	13	53.8	65.0
chronic arthritis	anaemia	ibd	2	13	61.5	65.0
mental illness	reflux disorders	chronic diarrhea	2	13	0.0	61.5
mental illness	neuropathic pain	menopausal and perimenopausal	2	13	0.0	40.0
chronic diarrhea	reflux disorders	anaemia	2	13	53.8	65.0
insomnia	reflux disorders	anaemia	2	12	43.3	62.0
chronic airway diseases	reflux disorders	chronic diarrhea	2	12	50.0	62.0
chronic arthritis	reflux disorders	chronic diarrhea	2	12	58.3	62.0
epilepsy	neuropathic pain	chronic arthritis	2	12	41.7	62.0
menopausal and perimenopausal	reflux disorders	chd	2	12	62.0	62.0
epilepsy	chronic arthritis	insomnia	2	12	41.7	62.0
neuropathic pain	chronic arthritis	heart failure	2	12	62.0	62.0
neuropathic pain	chronic arthritis	osteoporosis	2	12	43.3	62.0
chronic pain conditions	insomnia	neuropathic pain	2	12	0.0	40.0
insomnia	chronic arthritis	chd	2	12	43.3	62.0
chronic diarrhea	reflux disorders	chronic pain conditions	2	11	45.5	58.0
ibd	reflux disorders	neuropathic pain	2	11	45.0	54.5
insomnia	menopausal and perimenopausal	neuropathic pain	2	11	0.0	54.5
hypertension	chronic pain conditions	insomnia	2	11	45.5	52.0
menopausal and perimenopausal	chronic arthritis	pvd	2	11	0.0	45.5
chronic diarrhea	reflux disorders	menopausal and perimenopausal	2	11	54.5	55.0
chronic airway diseases	insomnia	chronic diarrhea	2	11	54.5	55.0
coronary heart disease	reflux disorders	neuropathic pain	2	10	50.0	52.0
chronic diarrhea	reflux disorders	dysphagia	2	10	52.0	52.0
reflux disorders	chronic diarrhea	insomnia	2	10	0.0	52.0
insomnia	neuropathic pain	hypertension	2	10	0.0	52.0
insomnia	neuropathic pain	diabetes	2	10	0.0	52.0
coronary heart disease	reflux disorders	dysphagia	2	10	53.0	100.0
chronic constipation	dysphagia	dementia	2	10	53.0	52.0
diabetes	neuropathic pain	menopausal & perimenopausal	2	10	52.0	52.0
insomnia	reflux disorders	chd	2	10	50.0	52.0
mental illness	chronic arthritis	pvd	2	10	50.0	52.0
hearing loss	chronic arthritis	neuropathic pain	2	10	52.0	0.0
insomnia	chronic arthritis	heart failure	2	10	50.0	100.0
insomnia	chronic arthritis	hearing loss	2	10	0.0	52.0
mental illness	chronic airway diseases	chronic pneumonia	2	10	52.0	52.0
chronic constipation	reflux disorders	insomnia	2	10	52.0	52.0

Continued on next page

Table S13 – continued from previous page

Condition 1	Condition 2	Condition 3	Cluster	N Patients	Mortality %	Long Hospital Stay %
chronic constipation	reflux disorders	ibd	2	10	45.0	52.0

Table S14. Characteristics of clusters defined by shared disease trajectories. This table presents the distribution of unique patients shared all trajectories across identified clusters, stratified by sex and age groups ($<$ 45 and $\geq$ 45 years).

	Males				Females		
	$<$ 45 years	$\geq$ 45 years			$<$ 45 years	$\geq$ 45 years	
Clusters	1	1	2	3	1	1	2
Total Patients	549.0	2824.0	1557.0	633.0	1713.0	6101.0	4057.0
Unique Patients	301.0	728.0	525.0	320.0	491.0	1201.0	1009.0
Mean age ($\pm$ SD)	35.6 (5.8)	62.7 (10.2)	59.4 (9.6)	59.7 (9.7)	35.9 (6.1)	63.0 (11.3)	59.3 (10.8)
Autism, N (%)	26.0 (8.6)	45.0 (6.2)	26.0 (5.0)	29.0 (9.1)	30.0 (6.1)	24.0 (2.0)	22.0 (2.2)
Psychotropic Medications, N (%)	68.0 (22.6)	81.0 (11.1)	60.0 (11.4)	69.0 (21.6)	74.0 (15.1)	171.0 (14.2)	130.0 (12.9)
Hospitalisation, N (%)	198.0 (65.8)	669.0 (91.9)	430.0 (81.9)	292.0 (91.3)	327.0 (66.6)	1047.0 (87.2)	793.0 (78.6)
Long Hospital Stay I, N (%)	141.0 (46.8)	612.0 (84.1)	354.0 (67.4)	263.0 (82.2)	215.0 (43.8)	899.0 (74.9)	624.0 (61.8)
Long Hospital Stay II, N (%)	135.0 (44.8)	604.0 (82.9)	351.0 (66.8)	256.0 (80.0)	207.0 (42.1)	883.0 (73.5)	607.0 (60.1)
Mortality, N (%)	51.0 (16.9)	462.0 (63.5)	251.0 (47.8)	222.0 (69.4)	66.0 (13.4)	703.0 (58.5)	434.0 (43.0)
Mortality rate	1.1	3.9	2.7	4.2	0.7	3.5	2.4

Note: SD = Standard Deviation. Psychotropic Medications include antipsychotics, antiepileptics, antidepressants and anxiolytics. Long Hospital Stay I is defined as a hospitalisation lasting more than 4 days. Long Hospital Stay II is defined as a hospitalisation lasting more than 4 days but less than 90 days. Mortality Rate = (Number of Deaths / Total Person-Years)$\times$100. Person-years for each patient were calculated as the time from their first recorded event to either their date of death or the end of the study period.

SUPPLEMENTARY FIGURES

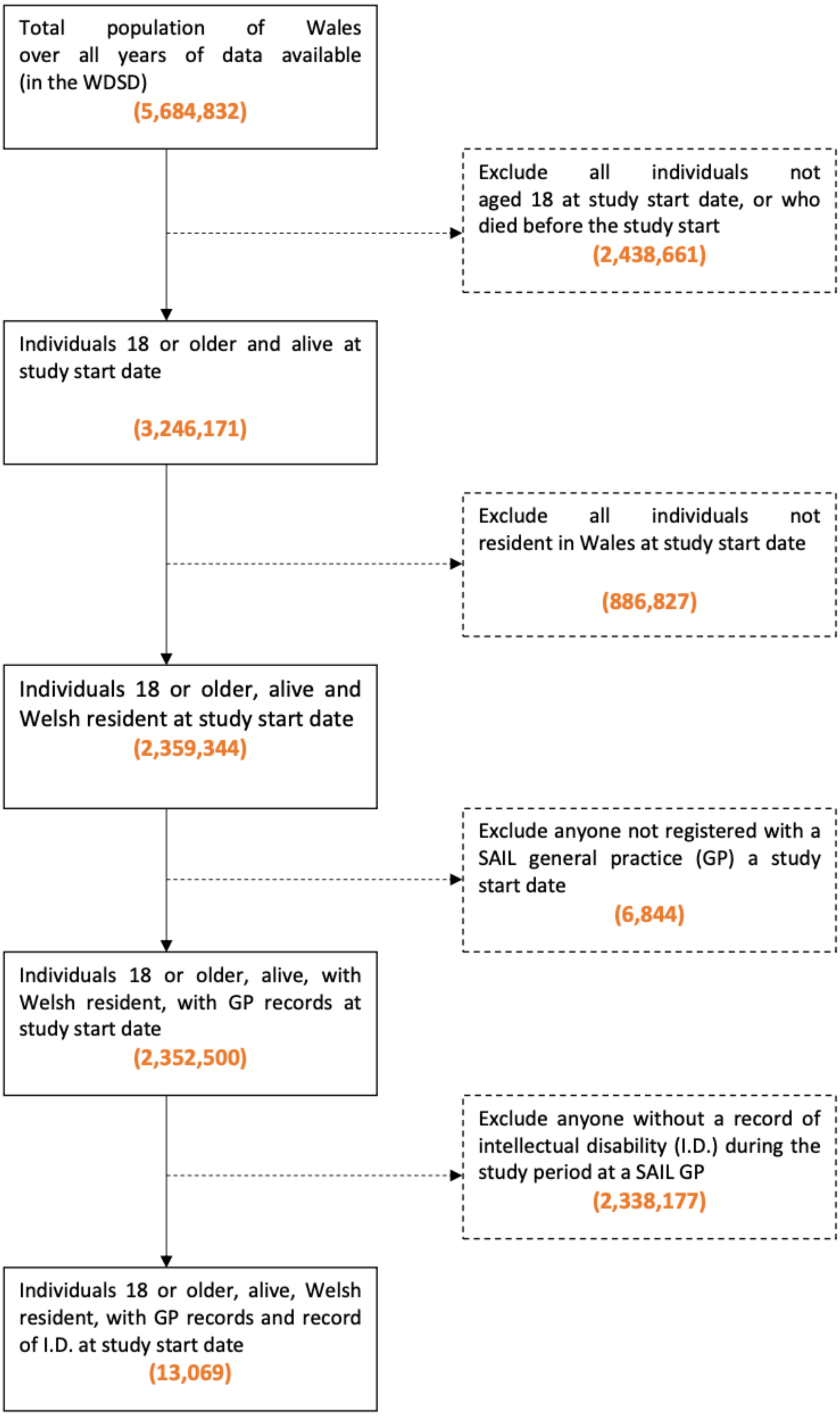

Figure S1: Consort Flow Diagram.

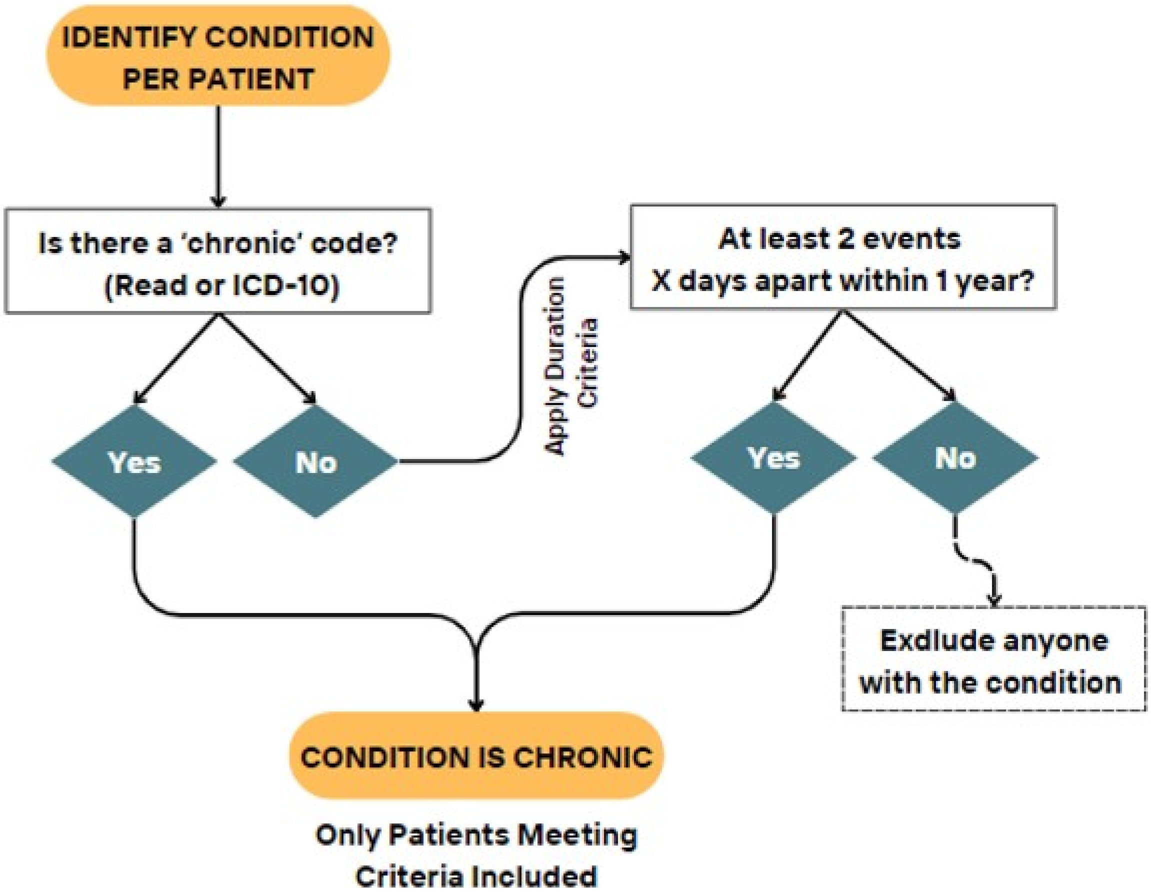

Figure S2: Defining chronic condition criteria.

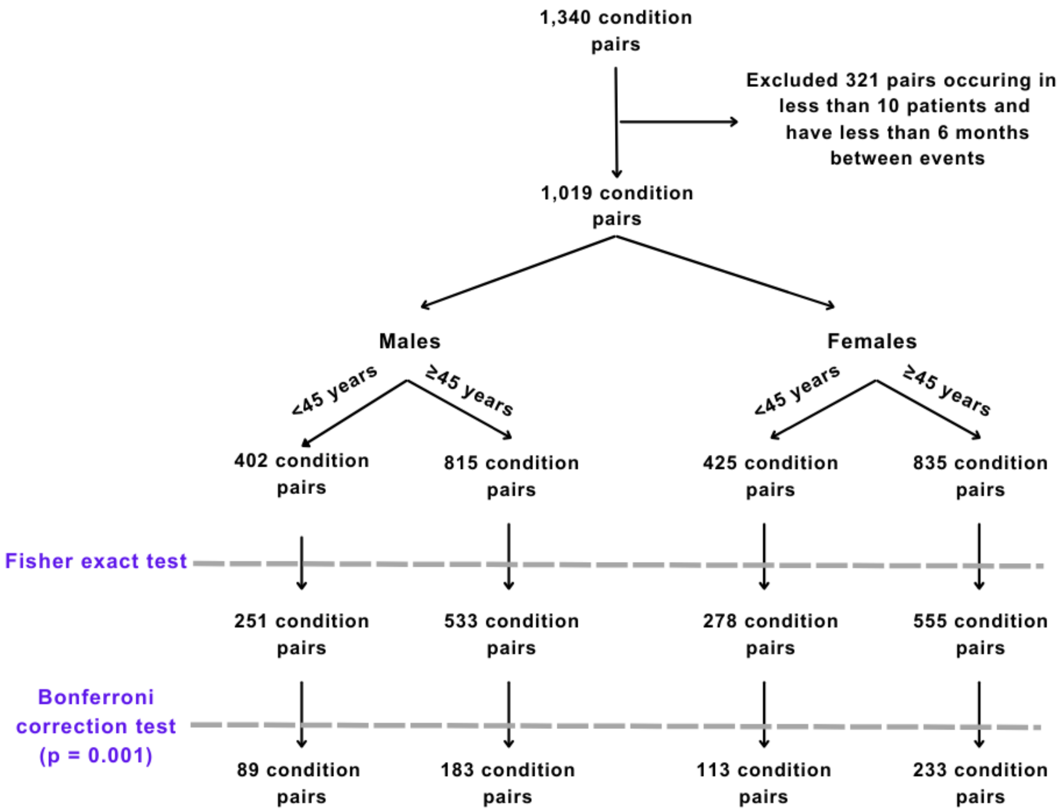

Figure S3: Long-term condition co-occurrences found in intellectual disability population data from 13,069 patients. 1,340 condition pairs were found to occur in the population; of these, a number of pairs were filtered out due to low frequency (N<10), or their events occur in less than six months period. After applying the Fisher exact test and Bonferroni correction the final condition pairs were found for both males and females stratified by age.
